# Long Term Rescue of the TSH Receptor Knock-Out Mouse – Thyroid Stem Cell Transplantation Restores Thyroid Function

**DOI:** 10.3389/fendo.2021.706101

**Published:** 2021-07-02

**Authors:** Rauf Latif, Risheng Ma, Syed A. Morshed, Bengu Tokat, Terry F. Davies

**Affiliations:** Thyroid Research Unit, Icahn School of Medicine at Mount Sinai and James J. Peters Veterans Affairs (VA) Medical Center, New York, NY, United States

**Keywords:** thyroid, stem cells, long term, transplantation, mouse ESC

## Abstract

The synergistic activation of transcription factors can lead to thyroid progenitor cell speciation. We have previously shown *in vitro* that mouse or human stem cells, expressing the transcription factors NKx2-1 and Pax8, can differentiate into thyroid neo-follicular structures (TFS). We now show that syngeneic mouse TFS when implanted into hypothyroid TSH receptor knockout (TSHR-KO) mice can ameliorate the hypothyroid state for an extended period. ES cells derived from heterozygous TSHR-KO blastocysts were stably transfected with Nkx2-1-GFP and Pax8-mcherry constructs and purified into 91.8% double positive cells by flow cytometry. After 5 days of activin A treatment these double positive cells were then induced to differentiate into neo-follicles in Matrigel for 21 days in the presence of 500μU/mL of TSH. Differentiated TFS expressing thyroglobulin mRNA were implanted under the kidney capsule of 4-6 weeks old TSHR-KO mice (n=5) as well as hind limb muscle (n=2) and anterior chamber of one eye (n=2). Five of the mice tested after 4 weeks were all rendered euthyroid and all mice remained euthyroid at 20 weeks post implantation. The serum T4 fully recovered (pre-bleed 0.62 ± 0.03 to 8.40 ± 0.57 µg/dL) and the previously elevated TSH became normal or suppressed (pre-bleed 391 ± 7.6 to 4.34 ± 1.25 ng/dL) at the end of the 20 week observation period. The final histology obtained from the implanted kidney tissues showed only rudimentary thyroid follicular structures but which stained positive for thyroglobulin expression. The presence of only rudimentary structures at the site of implant on these extended animals suggested possible migration of cells from the site of implant or an inability of TFCs to maintain proper follicular morphology in these external sites for extended periods. However, there were no signs of tumor formation and no immune infiltration. These preliminary studies show that TSHR-KO mice are a useful model for orthotropic implantation of functional thyroid cells without the need for thyroidectomy, radioiodine ablation or anti thyroid drug control of thyroid function. This approach is also proof of principle that thyroid cells derived from mouse ES cells are capable of surviving as functional neo-follicles *in vivo* for an extended period of 20 weeks.

## Introduction

**Figure 1:**
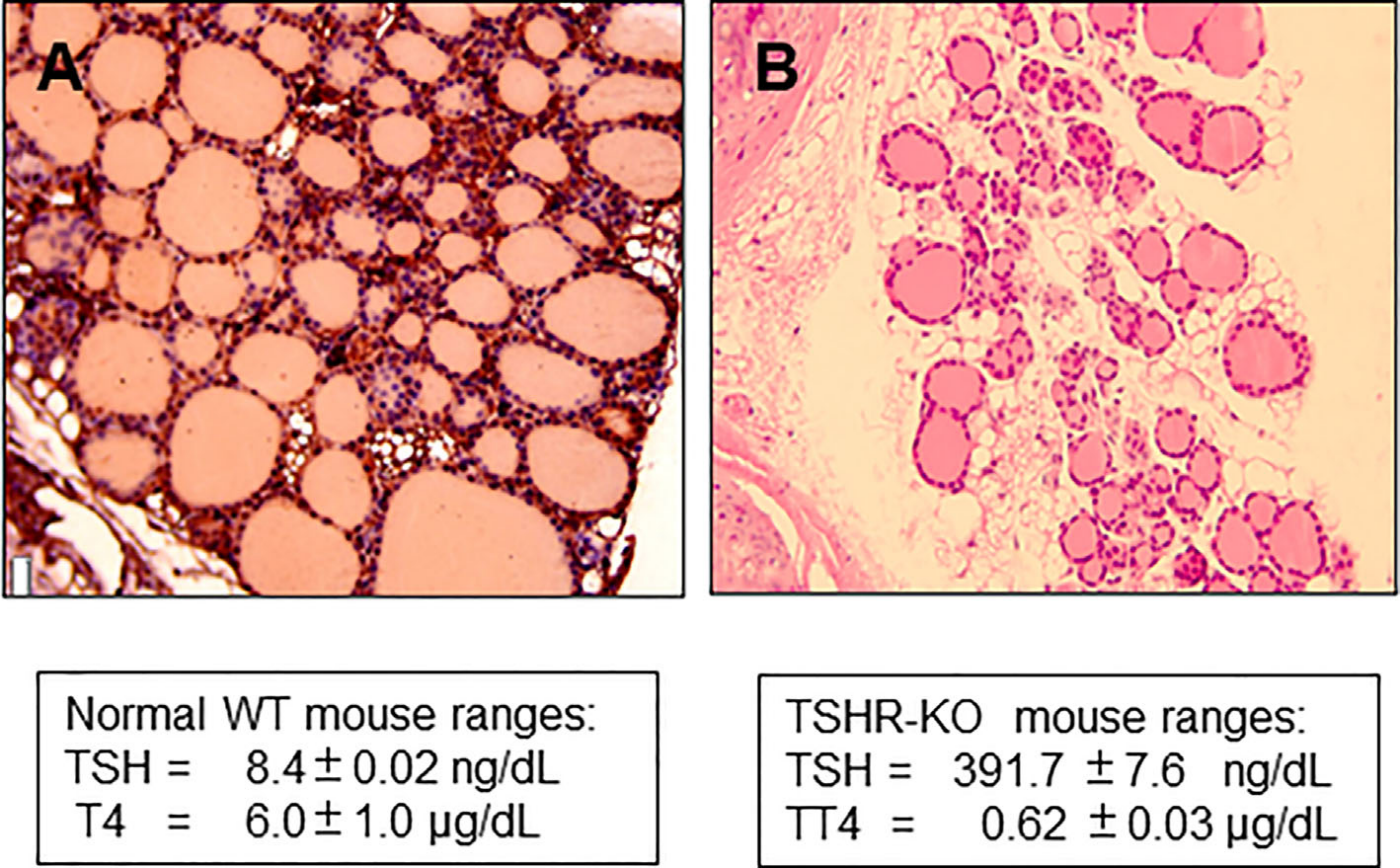
Histology of TSHR-KO thyroid. **(A)** shows the histological appearance of a 6 week normal mouse thyroid gland compared to **(B)** taken from a 6-week-old TSHR-KO mouse. The knockout mice have approximately 1/3 of the number of follicles in a normal mouse indicating that TSH and TSHR signaling is needed for post-natal development of the gland.

The TSH receptor (TSHR) is the major trophic activator of the thyroid gland and controls thyroid hormone synthesis and secretion. Although the TSHR is expressed in a variety of tissues its major physiologic role is in the thyroid follicular cell. The development of the hypothyroid TSH receptor knock-out (TSHR-KO) mouse in 2002 (1) opened a new *in vivo* line of investigation into TSH action and thyroid function which has been utilized by many investigators for almost 20 years. The TSHR-KO mice have a small thyroid gland indicating that development of the gland is not totally dependent on TSH, but the mouse is markedly hypothyroid with only rudimentary thyroid follicles (**Figure 1**), low thyroxine levels and very high serum TSH. These mice remain stunted unless weaned at 3 weeks and put on a thyroid replacement diet. Rendering such hypothyroid mice to be consistently euthyroid has not, however, been easy with the variable ability and availability of thyroid hormone replacement strategies which usually require a special, and sometimes unreliable diet, to be commissioned.

The synergistic activation of the transcription factors NKx2-1 and Pax8 in embryonic stem (ES) cells has now been shown to lead to thyroid progenitor cell speciation. We and others have previously shown (2, 3) that (ES) cells or induced pluripotent stem (iPS) (4) cells, expressing these two transcription factors, can differentiate into thyroid neo-follicular structures (TFS) with TSH stimulation. This development of functional thyroid cells from murine ES cells has now raised the option of their transplantation (2) and in the case of the TSHR-KO mouse, of maintaining long term euthyroidism by transplantation of syngeneic stem cells. To date, there is no published evidence that hypothyroid mice can be sustained for more than a few weeks using mouse ES cell derived thyroid follicular cells. This study was, therefore, initiated to see if transplanted mouse ES cells survived long term in their implanted sites and were able to rescue the hypothyroid TSHR-KO mouse by normalizing their thyroid function tests without resulting in tumors at the implantation site.

## Methods

### Mouse Models

SCID mice were purchased from Jackson Labs and maintained in a pathogen free environment. Our TSHR-KO colony has been described in detail previously and the genotype of all mice were confirmed (1). The TSHR-KO mouse line has a mixed background of C57/B6J-129. Mice were supplemented with T4 containing chow at a concentration of 100 ppm. Thyroid replacement was withdrawn 4 weeks before each experiment and each mouse was assessed by assay of serum total T4 and TSH prior to inclusion (see below). All transplantation studies involving SCID and TSHR-KO mice were approved by the Institutional Animal Care and Use Committee of the Icahn School of Medicine at Mount Sinai.

### Development and Characterization of Co-Expressing Mouse Embryonic Stem Cells

**Figure 2:**
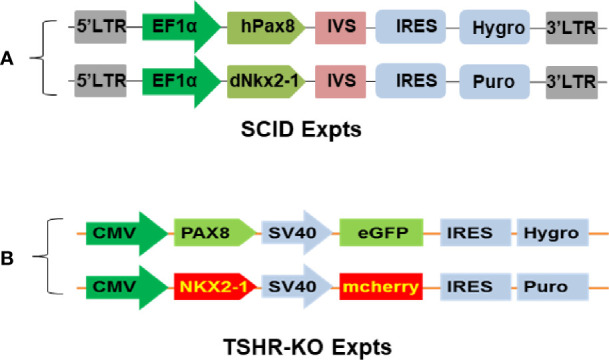
Establishment of syngeneic double positive mouse ES+/- cells. **(A, B)** represent the bicistronic lent viral vector constructs used for establishing double positive transfected mouse cells expressing Pax8 and Nkx2-1 with and without reporter genes.

**Figure 3:**
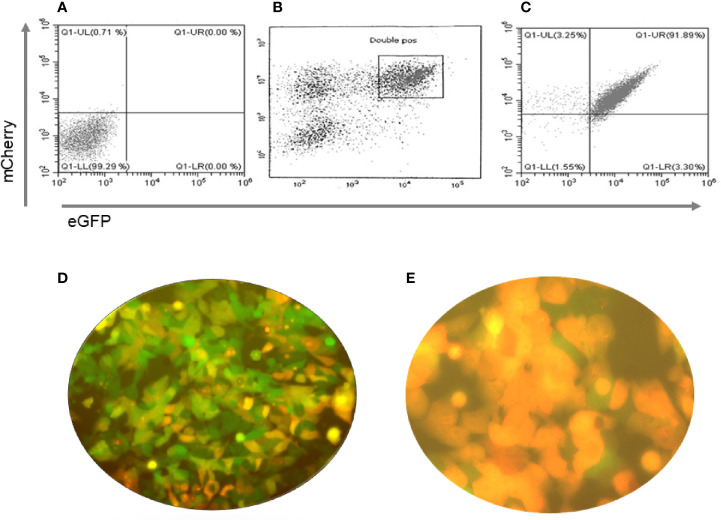
Purification of double positive mES cells. These figures show the flow cytometry profiles of pre and post sorted mouse ES +/- cells. **(A)** Unstained mES, **(B)** eGFP and mCherry double positive gated cells for sorting (boxed) and **(C)** Post sorted cell analysis indicated 91.89% purity. **(D, E)** Immunofluorescence images of the cells before and after sorting of the cells for GFP and mCherry fluorescence with the sorted cells being all double positive.

For short-term SCID mouse experiments we generated cells that overexpressed human Pax8 and dog Nkx2-1 in wild type mouse (m) ES cells (W9.5 ES) using the constructs described in **Figure 2A**. For the TSHR-KO mouse studies we used our previously reported TSHR+/− mES cell line derived from female TSHR-KO mouse (5). To generate heterozygous mES cells overexpressing human Pax8-GFP and Nkx2-1mCheery we co-transduced the cells with pEZ lentivirus bicistronic constructs **(** **Figure 2B** **)** driven by the CMV promoter. In both types of cells the stable clones were obtained by selecting the transduced cells with hygromycin and puromycin. Since these selected stable lines had both single transfected and/or low expressing cells along with double transfected cells, as indicated by the microscopic images (**Figure 3D**), we performed fluorescent activated cell sorting (FACS) using GFP and mCherry as markers to enrich the population for double positive cells (**Figure 3** **)**.

### Differentiation of ES Cells

**Figure 4:**
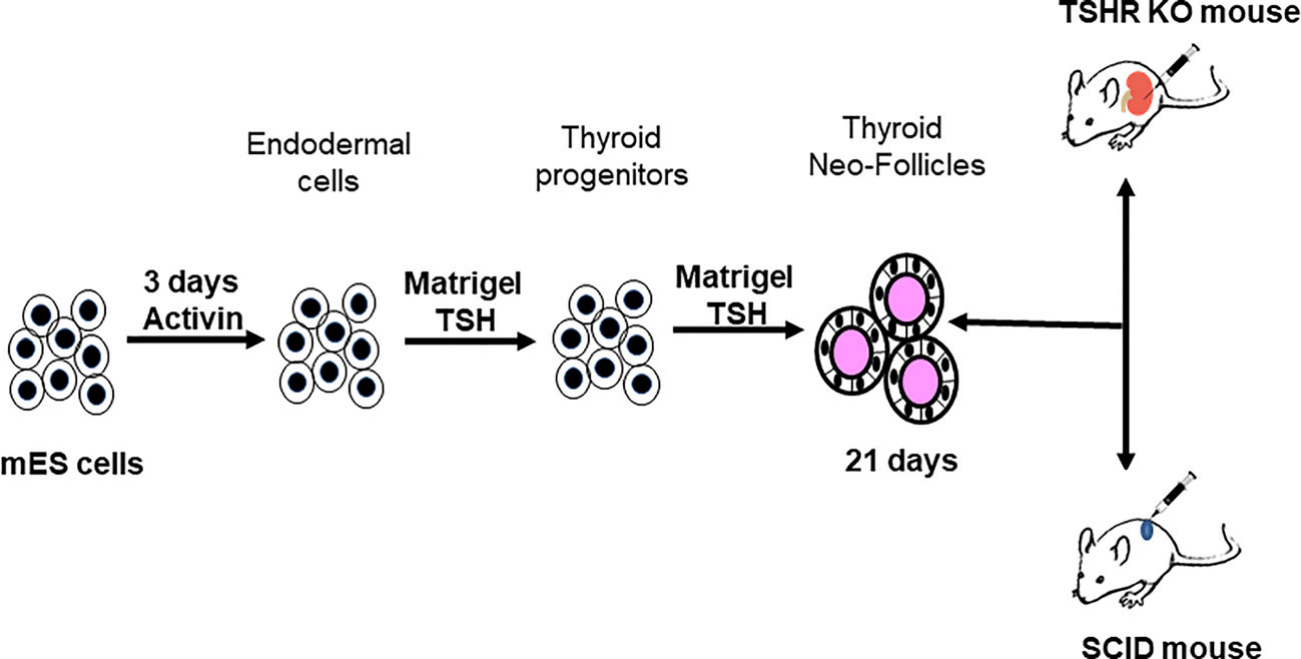
Protocol for thyroid cell differentiation. Stable mES+/- cells transfected with Pax8 and Nkx2-1 with and without tagged reporter constructs were first differentiated to form anterior endodermal cells by activin A treatment. These cells were then embedded in a Matrigel scaffold and treated for 21 days with TSH to differentiate into thyroid follicular cells (TFCs). The differentiated cells were recovered from the scaffold, washed, counted and resuspended in medium prior to injecting them either into SCID mice or TSHR-KO mice as detailed in **Figure 5**.

ES cells were differentiated into endodermal cells and moved further into thyroid follicular cells (TFCs) using the protocol schematically represented in **Figure 4**. This protocol included the derivation of endodermal cells from the naïve ES cells with 3 days of activin A at low concentrations (50-70ng/mL) in LIF minus mouse ES medium. The endodermal cells were then allowed to form into embryoid bodies (EBs) in low attachment plates overnight in LIF minus medium and further driven into the path of TFCs by reseeding them with TSH in Matrigel coated plates with continued treatment of TSH (500uU/mL) for 21 days as the only additional differentiation factor. Periodic sub-culturing into fresh Matrigel was used to maintain the differentiated state. We used female mice for this study.

### Cell Preparation and Transplantation of Cells

mES WT, mES WT- Pax+Nkx2+ and mES+/- PaxGFP+Nkx2-1mCherry cells were each maintained in Matrigel with TSH in 6 well plates for 21 days of differentiation as outlined in the detailed protocol **(** **Figure 4** **)**. Fully differentiated cells for each of the SCID and TSHR-KO protocols were suspended by gently pipetting them up and down and collecting into a 15mL polypropylene tube. The cells were washed once with 1X PBS pH 7.4 and pelleted by centrifuging at 1000rpm for 4 minutes at room temperature. The cell pellets were re-suspended in 50 μL of ES cell differentiation medium ready for injection.

For the initial short term experiments we used 4 week old SCID mice (n=5). 50µL of diluted cell suspension containing ~0.5x 10^6^ cells was then injected subcutaneously into the dorsal flank of the SCID mice. For the longer term experiments we transplanted cells under the kidney capsule of 4-6 week old TSHR-KO mice (n=5). 10 µL of a pellet suspension (corresponding to ~1-2 x 10^6^ cells) were transplanted under the right kidney capsule. For transplantation the mice were anesthetized by injecting 0.1mL/10gm body weight of an equal ratio mixture of ketamine and xylaxine anesthetic. Before transplantation the lateral site of the right abdomen was shaved, scrubbed with antiseptic solution and a vertical subcostal incision of the skin, muscle and peritoneum was made (approximately 2.0cm; dorsolateral). The kidney was exposed by a blunt dissection instrument and 10µL of cells were injected under the kidney capsule using a syringe. The abdominal wall was closed, and the outer skin was stapled. The animals were allowed to recover from the anesthesia by holding them on a warm pad and then transferred into clean cages. For implantation of cells into alternative sites of the TSHR-KO mice, we injected 50µl of diluted cell suspension with ~1-2x10^6^ cells into the posterior tibialis muscle (n=2) and anterior chamber of one eye (n=2).

### Thyroid Function Testing

T4 and TSH in the mouse sera were assessed using the Milliplex Map technique (Millipore RTHYMAG-30K). Serum samples were diluted 1:6 in assay buffer and incubated with fluorescent - coated magnetic beads to capture total T4 and TSH from the sample. Serum thyroglobulin (Tg) was detected using a commercial capture ELISA (Biomatik, Kitchener, Ontario, Canada). Serum samples were diluted 1:200 in PBS and run against a provided standard. The minimum detectable concentration was 13.1pg/ml. No T4 or TSH was measured in the serum of the SCID mice because these animals were neither thyroidectomized nor radioiodine ablated.

### Histology and Immunohistochemistry

**Figure 5:**
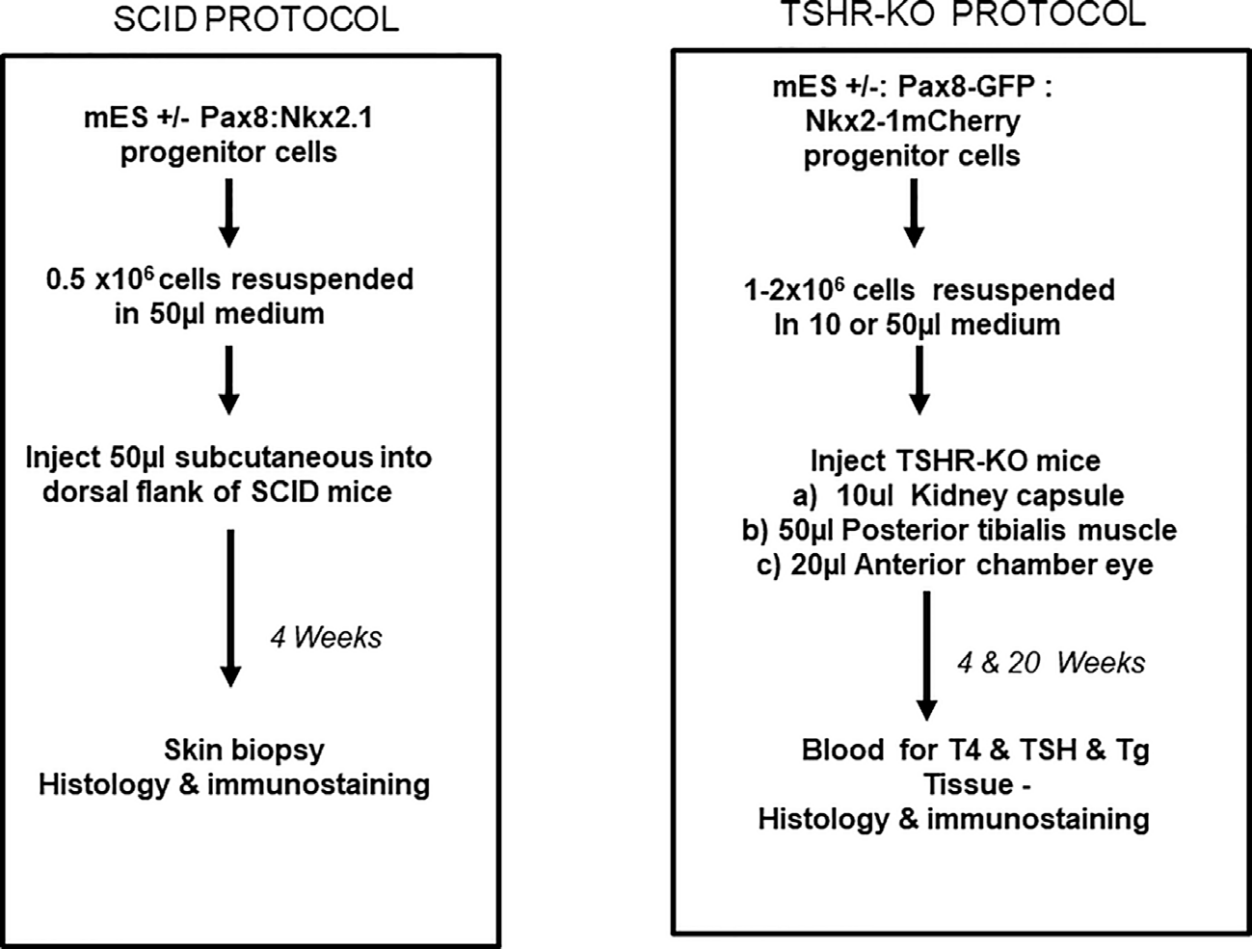
Transplantation protocols. Flow charts detailing the protocol used for the transplantation of the differentiated cells into either the SCID mice or into the TSHRO mice kidney/muscle/eye implantation sites.

Samples were collected from SCID mice or TSHR-KO mice at the end of 4 weeks or 20 weeks respectively, as indicated in **Figure 5**. Samples were fixed in 10% buffered formalin in 0.9% NaCl and then embedded in paraffin and 5 micron sections of the tissue was obtained for H & E staining and immunocytochemistry. Images of the stained H & E slides were acquired using bright field illumination on an inverted Nikon TE2000S microscope. To demonstrate the presence of thyroglobulin (Tg) in sections of implanted kidney from TSHR-KO mice, we used a high titer polyclonal mTg rabbit antibody that was generously provided by Dr. Peter Arvan, Michigan, USA (6). The tissue sections were incubated with this anti-mTg polyclonal antibody diluted 1:100 in staining buffer containing 0.3% triton X100 and incubated overnight at 4°C. After washing the sections thoroughly with PBS, the primary antibody was detected using an anti-rabbit IgG conjugated with Alexa 488 (1:200) (Cell Signaling Technology, Danvers, MA). Control sections with only anti rabbit conjugated Alexa488 were processed in parallel. All fluorescent images were also obtained on a Nikon TE 2000S microscope.

### Gene Expression for Thyroid Markers by qPCR Gene Expression

For gene expression by qPCR, total RNA was extracted (RNeasy) including removal of genomic DNA by deoxyribonuclease treatment following the recommended protocol. Five micrograms of total RNA were then reverse transcribed into cDNA (SuperScript III system). All qPCRs were performed using the Step OnePlus Real-time PCR system (Applied Biosystems, Foster City, CA). The reactions were established with 10 μL of SYBR Green master mix (Applied Biosystems, Foster City, CA), 0.4 μl (2μM) of sense/anti-sense gene-specific primers, 2 μl of cDNA and DEPC-treated water to a final volume of 20 μl. The PCR reaction mix was denatured at 95°C for 60 seconds before the first PCR cycle. The thermal cycle profile was used is as follows: denaturizing for 30 seconds at 95°C; annealing for 30 seconds at 57- 60°C (dependent on primers) which was determined by the melt curves of each primer; and extension for 60 seconds at 72°C. A total of 40 PCR cycles were used. For each target gene, the relative gene expression was normalized to that of the glyceraldehyde-3-phosphate dehydrogenase (GAPDH). Data are presented as fold change in relative gene expression and are from two independent experiments in which all sample sets were analyzed in triplicate. The thyroid specific primer sets used are given in **Supplementary Table 2** and were as previously published (2).

### Data Analysis

All graphs and statistical analyses were performed using GraphPad Prism Version 6.04. Mean and SD values were used from duplicate measurements.

## Results

### Thyroid Progenitor Cells for Transplantation

We have previously described the transformation of transfected WT mES cells into thyroid progenitor cells and their expression of thyroid specific genes (7) and these cells were used for the SCID experiment. In contrast, the TSHR-KO heterozygous cells were purified using GFP and mCherry expression as markers. By gating on these double positive cells, we obtained nearly 92% purity of these cells **(** **Figures 3A–E** **).** These purified and fully differentiated TFCs were then used for the longer-term implantation experiments in TSHR-KO mice.

### The SCID Mouse Transplantation Model

**Figure 6:**
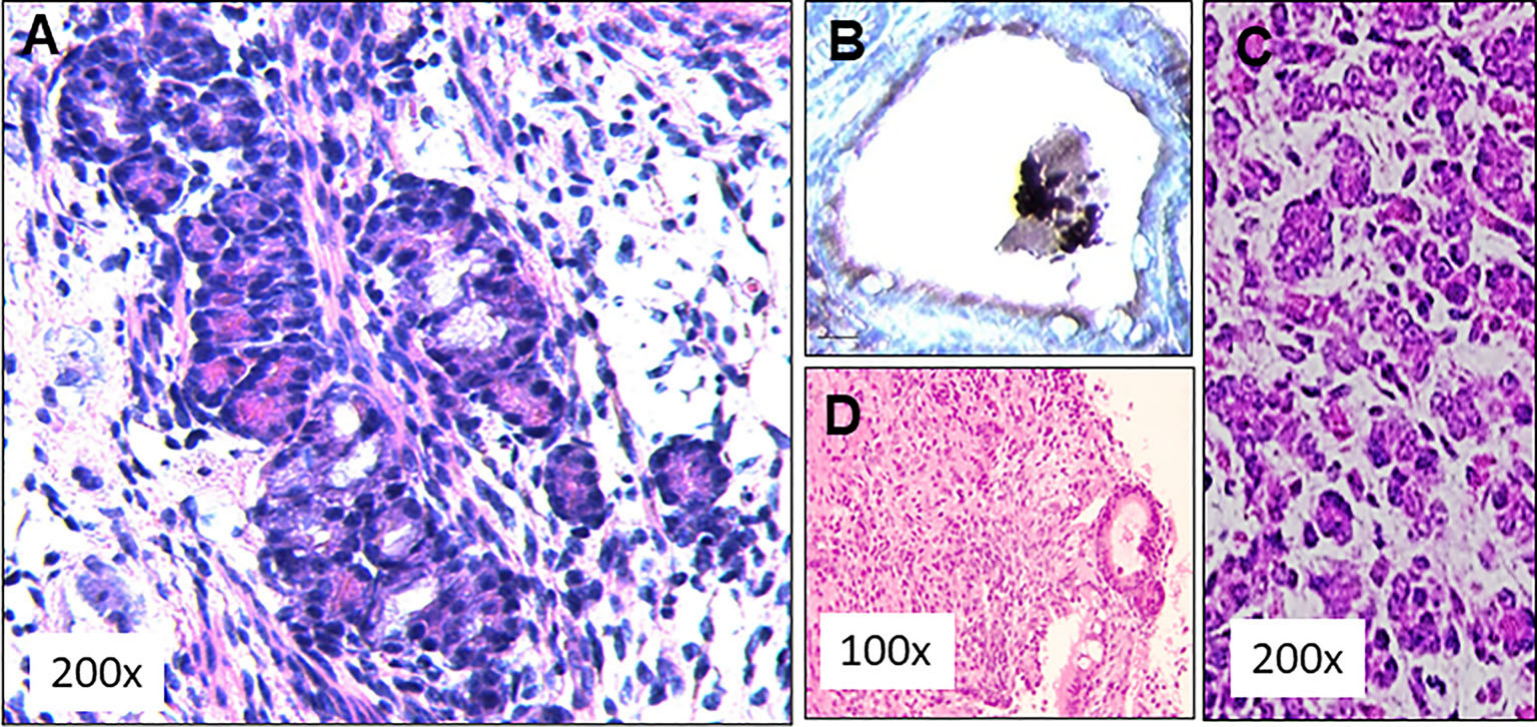
SCID mouse transplant histology. **(A)** Subcutaneously implanted dermal tissue was analyzed histologically for the presence follicular structures. A large number of rudimentary follicular like structures were observed (H&E staining). **(B)** These follicular structures were stained for the presence thyroglobulin (Tg) using an anti Tg antibody. Intracellular Tg was observed in peripheral follicular cells but the central colloid remained negative for Tg, **(C)** This picture from a 13 day normal fetal thyroid shows some similarities to the histology obtained in the 4 week transplant samples. **(D)** Histological staining of the tissue from 3 of these mice showed undifferentiated cells between thyroids like structures indicating anaplastic tumor initiation at the implanted sites.

Initial experiments for the feasibility of transplanting our ES cell derived mouse thyroid cells were performed in SCID mice to reduce the likelihood of immune rejection. We have used this approach previously for human thyroid organoid development (8). The ES cells were differentiated as outlined in **Figure 4** and then injected subcutaneously into 5 SCID mice as described in **Figure 5**. 4 weeks after subcutaneous delivery of 5 X 10^6^ cells the dermal histology indicated survival and thyroid follicle formation in the mouse dermis in each of the mice (**Figure 6A**). Immunohistochemical staining of implanted tissues with specific Tg antibody showed intracellular staining of Tg within the follicular cells. However, no Tg staining was observed within the central colloid in these follicular structures (**Figure 6B**). The histology was reminiscent of a fetal mouse thyroid (**Figure 6C**). However, of the initial 5 mice used for this experiment, 3 of the mice also showed the development of undifferentiated/anaplastic tumors at the injection site by 6 weeks (**Figure 6D**). We interpreted this to indicate the need for ES cell purification prior to transplantation and proceeded to prepare ES cells purified by using the GFP and mCherry transfected cells.

### Purified Cells for TSHR-KO Mouse Transplantation

**Figure 7:**
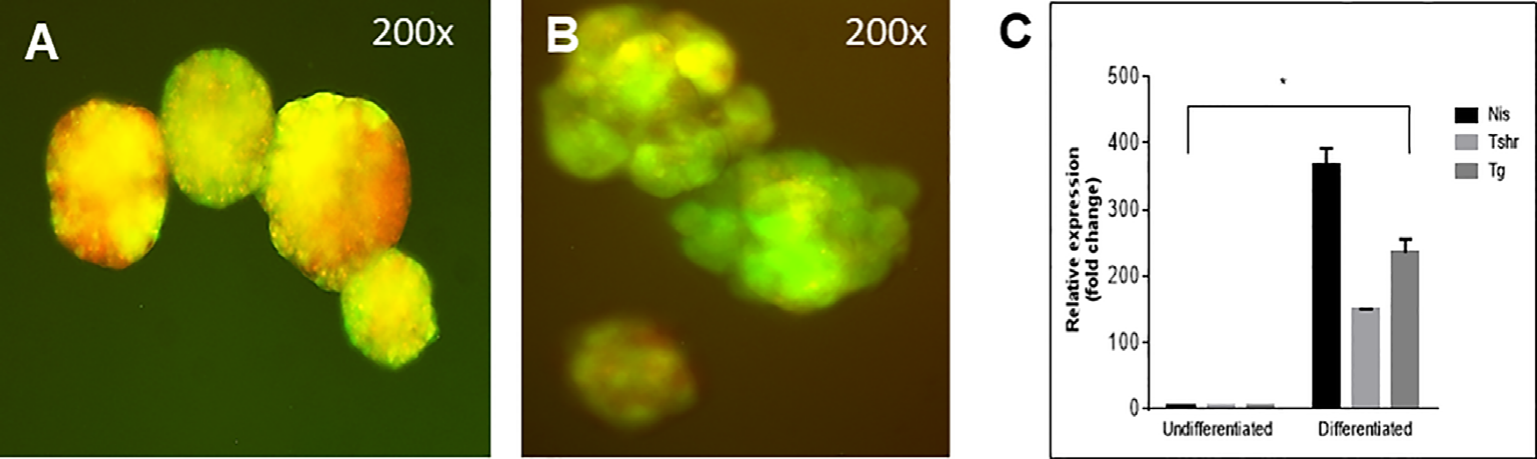
Follicle structure and gene expression of labelled cells. **(A)** mES+/- cells formed embryoid bodies (EBs) when cultured in low attachment plates and **(B)** further differentiated into thyroid follicles in embedded Matrigel as observed with overlapping expression (yellow images) of Pax8-GFP and Nkx2-1mCherry. **(C)** qPCR data showed significantly enhanced expression of Tg, TSHR and NIS in these thyroid follicular cells compared to the undifferentiated cells (*P < 0,001).

The differentiation of the EBs was monitored by their ability to form follicular-like structures. As previously observed (2) these EB derived follicular cells at the end of 21 days were first monitored for the simultaneous expression of Pax8-GFP and Nkx2-1-mCherry shown by overlapping yellow fluorescence in the EBs and follicular-like thyroid cells **(** **Figures 7A, B** **)**. Gene expression analysis indicated that these were differentiated thyroid cells. They expressed the major thyroid gene markers including thyroglobulin (Tg), thyroid stimulating hormone receptor (Tshr) and the sodium iodide symporter (NIS) which confirmed that these cells were, as we have previously defined (9), differentiated progenitor cells **(** **Figure 7C** **).**

### Prolonged *In Vivo* Studies of TSHR-KO Mouse Transplants

**Figure 8:**
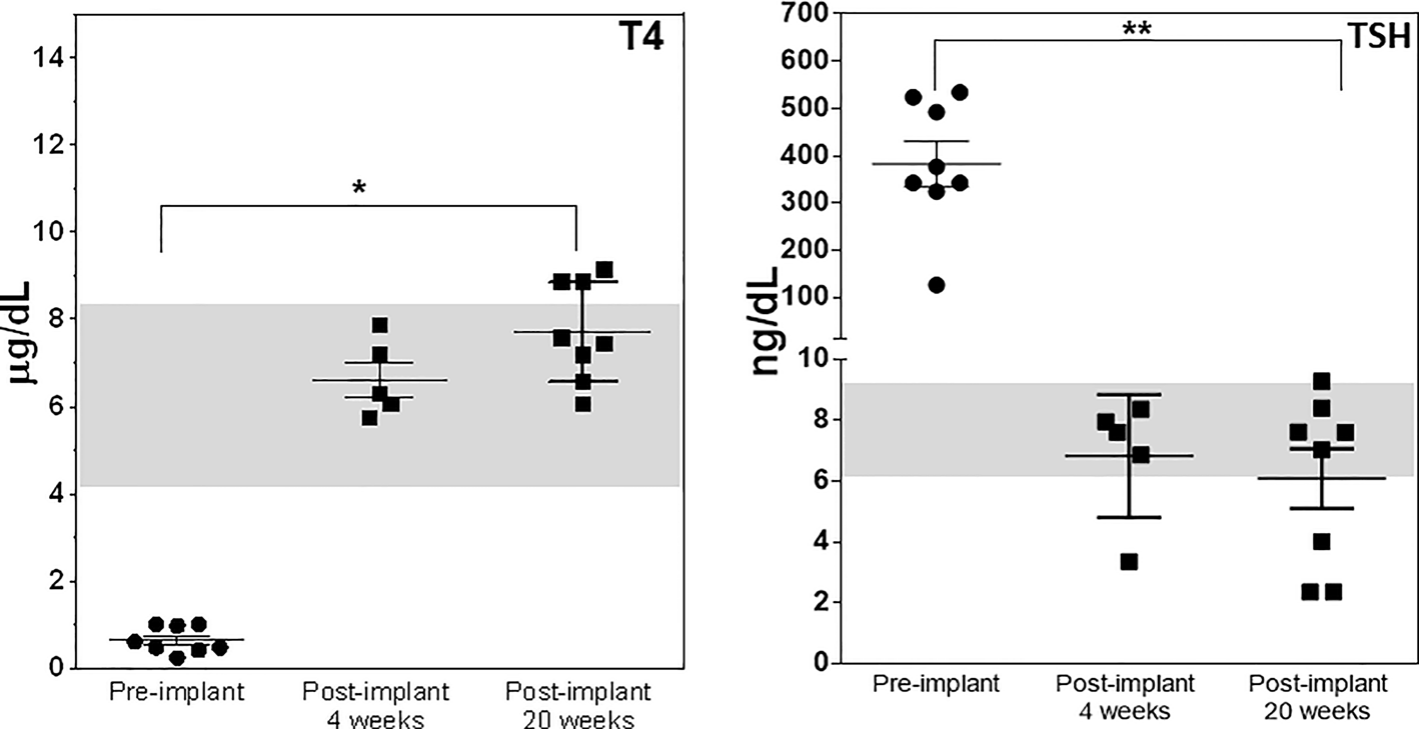
Thyroid function post transplantation. Total T4 (left panel) and TSH (right panel) were measured in the serum of mice implanted with cells under the kidney capsule at 4 and 20 weeks and in the muscle and eye at just 20 weeks using the multiplex bead assay as described in Materials and Method. Significant serum T4 elevation (*p < 0.01) and TSH suppression (**p < 0.001) was observed in all animals that survived until 20 weeks.

**Figure 9:**
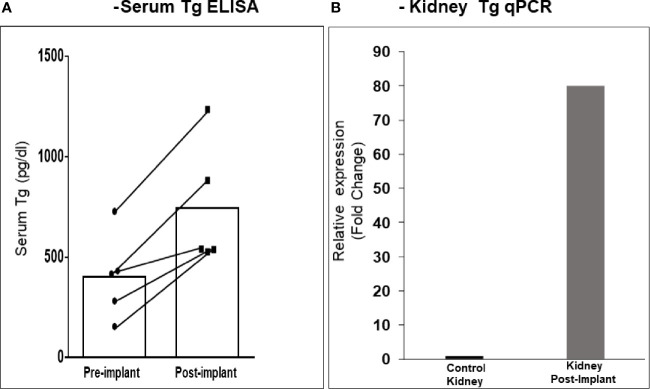
Serum Tg and renal Tg mRNA in transplanted mice. **(A)** The kidney implanted mouse sera showed elevated levels of circulating Tg compared to the pre-bleed sera when measured by ELISA. **(B)** Kidney tissue from mice at 20 weeks was analyzed by qPCR for Tg mRNA expression. As indicated a ~ 80-fold increase in expression was observed in kidney implanted animals versus control kidney. Since the tissue was from a limited number of animals (n = 3) a statistical evaluation could not be performed.

After 21 days of *in vitro* differentiation the double positive differentiated cells were harvested and prepared for implantation into genotype confirmed, and thyroid function tested, TSHR-KO mice as described in **Figure 5**. 1-2 x 10^6^ cells were implanted beneath the kidney capsule or the hind leg muscle or into the anterior chamber of one eye. We observed improved thyroid function in the tested mice by 4 weeks (n=5) and by 20 weeks post implantation (n=8) – 5 kidney implanted, 1 muscle (1 died) and 2 eye implanted mice – all of which showed normalization of their T4 and TSH levels (**Figure 8**). In keeping with this response was an increase in detectable serum Tg in the serum samples available (**Figure 9A**). The remarkable changes in the thyroid function tests of the individual mice are also shown in **Table 1**.

**Table 1:** Individual thyroid function tests before and 20 weeks post implantation.

Pre-implant T4 (µg/dL)	Post-implant T4 (µg/dL)	Pre-implant TSH ng/dL	Post-implant TSH ng/dL
0.47	8.86	324.27	8.39
0.24	7.42	534.48	2.35
0.6	7.17	523.83	9.26
0.4	6.05	342.44	7.61
1	8.86	492.32	7.01
0.47	6.56	126.23	2.36
0.96	7.57	342.44	7.61
1	7.5	376.41	4.01

### Transplant Analysis

**Figure 10:**
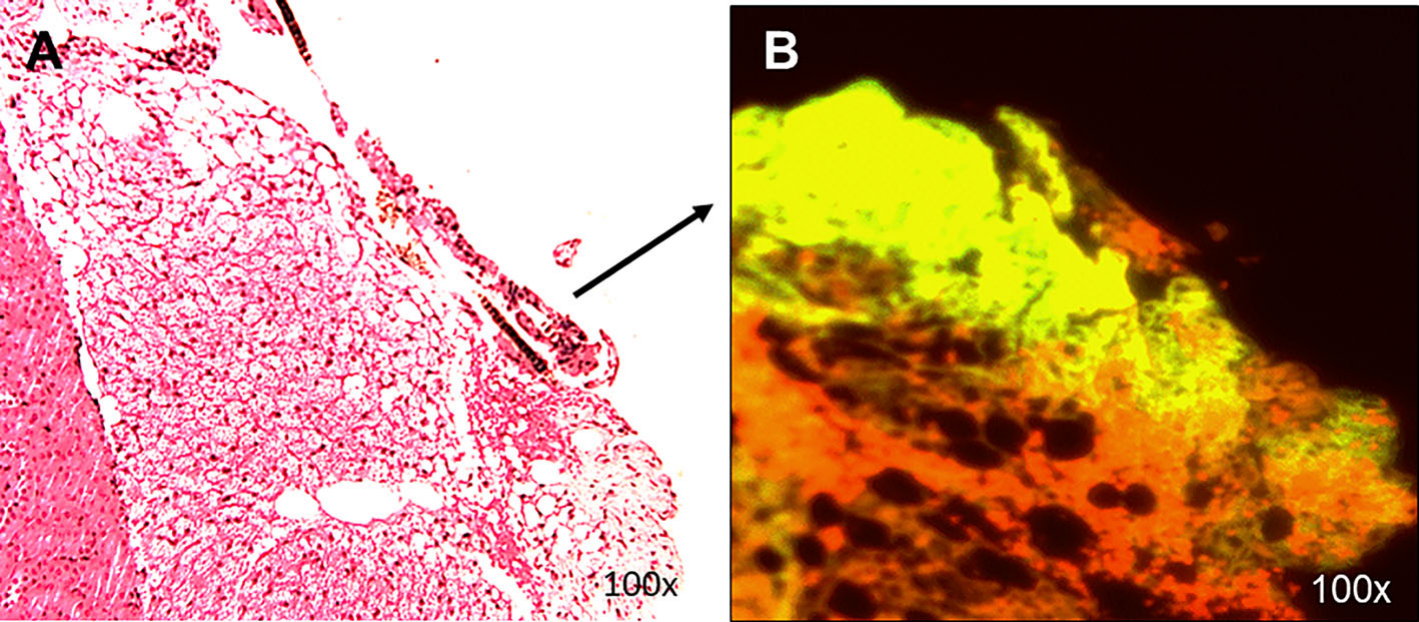
Histology of renal transplants under the capsule. Cells implanted under the kidney capsule were analyzed histologically for thyroid follicles **(A)**. The presence of follicular structures was further confirmed by **(B)** showing GFP and mCherry overlap fluorescence of the implanted region with the expression of yellow fluorescence. This sample of a localized transplant was from 4 weeks post-transplant.

**Figure 11:**
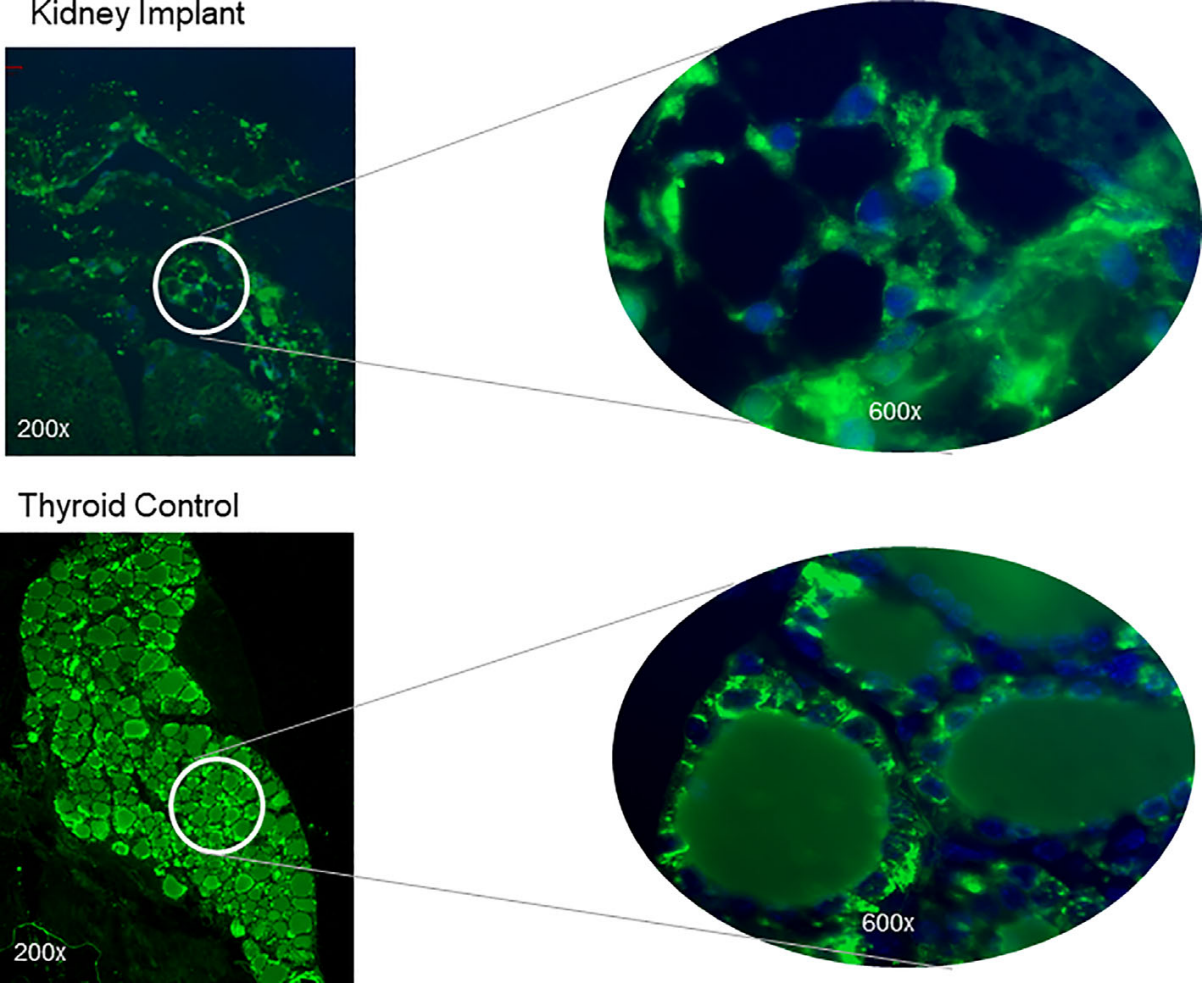
Tg expression in thyroid follicles at 20 weeks. Kidney sections of the implanted tissue were analyzed for expression of TG protein by using a polyclonal anti-Tg antibody. The top panel shows magnified images showing intracellular staining of Tg in the thyroid follicles without strong staining in the central colloid. The nuclei were stained blue with Dapi. A control normal thyroid section stained at the same time following the same protocol is shown below. Strong intracellular staining of the follicular cells was observed.

Histological analyses of 2 kidneys are shown in **Figures 10** and **11**. Confirmation of thyroid follicle formation and survival was found in each. In **Figure 10** the transplant is shown with its auto fluorescence from the transfected constructs. Green and red fluorescence from the GFP and mCherry combined to give an overlap of yellow fluorescence. Rescued follicles (**Figure 11** **)** showed positive intracellular staining for Tg expression and could be compared with normal mouse thyroid. The presence of Tg gene expression by qPCR was also confirmed in a limited number of available kidney tissues (**Figure 9B**). However, the volume of surviving thyroid tissue was small and mostly less well developed than seen earlier in the short term studies. No tumor formation was observed in any of the samples (n=8).

### Other Sites of Stem Cell Transplantation

In order to examine the feasibility of using alternative sites where differentiated thyroid stem cells could survive in the TSHR-KO mouse we also implanted cells into the posterior tibialis muscle (n=2 but 1 died) and into the anterior chamber of the eye (n=2). T4 and TSH measurements in these animals also showed increased T4 and suppressed TSH after 20 weeks of the study as indicated in **Table 1**. However, we were not able to locate residual thyroid follicles in the muscles nor in the eye on histological evaluation despite the maintenance of thyroid function.

## Discussion

The genesis of potential stem cell therapy for thyroid replacement has followed advancements in laboratory techniques for the control of stem cell cultures and differentiation into functional thyroid cells. This area of thyroid stem cell biology has progressed with remarkable speed and to such an extent that expectations are now so high that human thyroid transplantation is thought to be soon a reality. But much caution is still required. While the procuring of differentiated thyroid cells is now well characterized there remain many hurdles to their long term use *in vivo*. To date only short-term studies of such transplanted cells have been reported in mice with radioiodine ablated thyroid glands (10) and so our hypothesis was that long term survival of such cells was possible but needed to be verified. Since we have previously described a hypothyroid mouse model with no functional TSH receptors (TSHR-KO mouse) (1) this seemed an ideal model in which to initiate such studies.

The TSHR-KO mouse is a good model of hypothyroidism which is useful for studying treatment protocols to correct the deficit (1). This mouse has only a small rudimentary thyroid, but it develops in the appropriate place and expresses a reduced number of follicles showing that thyroid follicle development and formation is certainly not TSH dependent (**Figure 1**) (11). Mice replaced with T4 from birth develop normally despite the TSHR not being expressed in a variety of tissues including the brain (1, 11). However, careful investigation has revealed subtle deficits in bone formation (12, 13) and brain function (14) even in heterozygous mice indicating that the TSHR is involved in modulating a wide variety of extra-thyroidal activities. Although the TSHR-KO mouse has no functional TSH receptors it expresses the same immune responses to immunization with TSHR antigen as do normal mice (15) indicating that this model remains tolerant to the TSHR (16). Most importantly, such information indicates that the TSHR-KO mouse should be able to accept syngeneic TSHR expressing stem cell derived thyroid follicular cells in the protocol we describe here.

A number of investigators have demonstrated short term survival of transplanted thyroid cells including our original description of thyroid “organoids” (8, 17) which formed neo-follicles from human thyroid primary monolayer cells (17) but had limited growth potential. With the development of protocols for thyroid cell differentiation from ES and iPS cells (2–4), short term survival of transplanted thyroid cells was soon demonstrated and the formation of thyroid follicles under the kidney capsule was well illustrated with improved thyroid hormone levels in thyroid ablated animals (18, 19). In the current studies we achieved similar success but over a longer period of time. The remarkable thyroid function shown in the current experiments with the TSHR-KO mice remaining euthyroid at 20 weeks indicates the potential longevity of this approach in contrast to the tumor formation we found earlier in transplants of unpurified cells into SCID mice. For the TSHR-KO mice we used highly purified ES cells selected for Pax8 and NKx-2.1 expression and we have seen no tumor formation to date. This further suggests that transplantation of these differentiated cells needs to be devoid of any undifferentiated ESCs to prevent tumor formation in. However, the caveat to this survival in an extra thyroidal cellular environment was that few cells were found at the site of implantation suggesting that the extracellular environment or extracellular matrix in which the differentiated cells are made to survive is likely to have a significant impact on thyroid cell behavior. The specific differentiation pathways generated *in vitro* clearly enriches certain precursors and achieving a similar effect *in vivo* is challenging. Though the transplanted mice did survive in their restored euthyroid state, it requires further study to show that immune privileged sites are necessary and whether the cells found additional niche sites such as the thyroid glands themselves. Studies have shown that when pancreatic beta cells were transplanted into the anterior chamber of rabbits that they survived well (20) and similarly cardiac stem cells in muscle also survive when transplanted into damaged heart muscle (21).

The fact that the histological studies showed only rudimentary follicle formation by 20 weeks despite the continuation of normal thyroid function strongly suggested that transplanted cells either failed to survive adequately, which would not explain the maintenance of thyroid function or were transported to additional niches within the body of the mice which now require cell tracing. It is not uncommon for stem cells implanted in one site of the brain to then migrate to other areas of the brain to establish functional recovery (22). Although cell-cell and cell –ECM interaction and concentrations of soluble factors are what hold cells in their steady state in their microenvironment, we can speculate that such changes would have been triggered in our long term implanted mice which need further careful examination. The TSHR-KO mouse is considered to have developed peripheral tolerance to the TSHR as assessed by its normal immune response to the TSH receptor which did not differ from normal control mice (16). Thus, the failure of significant survival of the transplants also raises the issue of whether natural autoimmunity played some part in this phenomenon. Since we did not see any infiltration of lymphocytes in our histological sections, nor did immunostaining of T and B cells reveal any significant number of these immune cells at the transplant location (data not shown), then this would appear to be an unlikely cause. Nevertheless, examining the immune response to the transfected ES cells requires further careful evaluation.

## Data Availability Statement

The raw data supporting the conclusions of this article will be made available by the authors, without undue reservation.

## Ethics Statement

The animal study was reviewed and approved by Institutional Animal Care and Use Committee of the Icahn School of Medicine at Mount Sinai.

## Author Contributions

RL: Carried out the experiments, analyzed the data, and wrote manuscript. RM: Helped in experiments and manuscript writing. SM: Critical reading of manuscript. BT: Helping in experiments. TD: Data analysis and writing of manuscript. All authors contributed to the article and approved the submitted version.

## Funding

This work was supported in part by National Institute of Health (NIH) grant DK069713, the Segal Family Endowment, and the Veterans Administration Merit Award Program (to TD).

## Conflict of Interest

TD is a member of the Board of Kronus Inc, Star, and Idaho.

The remaining authors declare that the research was conducted in the absence of any commercial or financial relationships that could be construed as a potential conflict of interest.
