# Supplementary material for: Long Term Rescue of the TSH Receptor Knock-Out Mouse – Thyroid Stem Cell Transplantation Restores Thyroid Function
Source: Front Endocrinol (Lausanne). 2021 Jul 2;12:706101. doi: 10.3389/fendo.2021.706101 (PMC8283971; doi:10.3389/fendo.2021.706101)
Supplement: Supplementary file 1 [file Table_1.docx]

**Supplementary Tables**

**Supplementary Table 1: Antibodies used in the study.**

| **Antibody Detail** | **Species** | **Source** | **Cat No** | **Dilution** | **Reference** |
| --- | --- | --- | --- | --- | --- |
| Polyclonal Tg antibody | Rabbit | Gifted by Dr Peter Arvan  [University of Michigan Medical School](http://www.med.umich.edu/intmed/endocrinology/arvanlab/) | NA | 1:200 | Ref 6 |
| Anti-rabbit IgG (H+L), F(ab')2 Fragment (Alexa Fluor® 488 Conjugate) | Goat | Cell Signaling Technology,  Danvers, MA | 4412 | 1:1000 | NA |

NA= Not Applicable

**Supplement Table 2: List of primers**

| **Gene Symbol**  **(Ref Seq ID)** | **Amplicon Length** | **Direction** | **Start** | **Sequence** |
| --- | --- | --- | --- | --- |
| GAPDH (NM_008084) | 97 | Forward | 64 | CCCTTGAGCTAGGACTGGATAA |
|  |  | Reverse | 139 | GGGCTGCAGTCCGTATTTATAG |
| Tg  (NM_009375) | 102 | Forward | 1260 | AGGAACTCTTTGTTGACTCTGG |
|  |  | Reverse | 1340 | CTGCTCTGATGGCTTCTCTTAG |
| Tshr  (NM_011648) | 109 | Forward | 1407 | CTGCTAACCAGCCACTACAA |
|  |  | Reverse | 1494 | GGTCTACAGAGGCAATGAGAAG |
| Nis  (NM_053248) | 103 | Forward | 751 | GTGCTCAGTCTCGCTCAAA |
|  |  | Reverse | 833 | GGGAGCCACCTACTACAAATG |
